# Male-male lethal combat in the quasi-gregarious parasitoid *Anastatus disparis* (Hymenoptera: Eupelmidae)

**DOI:** 10.1038/s41598-017-11890-x

**Published:** 2017-09-19

**Authors:** Peng-Cheng Liu, Jian-Rong Wei, Shuo Tian, De-Jun Hao

**Affiliations:** 1 Center for the Sustainable Forestry in Southern China, Nanjing Forestry University, Nanjing, Jiangsu Province China; 2 The College of Forestry, Nanjing Forestry University, Nanjing, Jiangsu Province China; 3 The College of Life Science, Hebei University, Baoding, Hebei Province China

## Abstract

Most animals employ aggressive behaviours to acquire resources such as food, territory and mates. Although mating is important for males, which typically exhibit competitive behaviours to gain mating opportunities, they generally tend to avoid conflict escalation; while extreme combat also occurs in some species and results in death. In this study, male-male lethal combat behaviour in *Anastatus disparis* was examined (Hymenoptera: Eupelmidae) by investigating the characteristics of fighting and the factors that influence fighting intensity in this species. Male fight intensity in *A*. *disparis* increased with both competitor density and female presence, while it was not influenced by the relatedness among male competitors. By comparing the frequency of received attacks between injured and non-injured males, we found that the former were more vulnerable to attack. In contrast to death due to lethal attack, death that occurs as a result of *A*. *disparis* combat may be the cumulative effect of injuries sustained over repeated competitive encounters. Combined with the biological characters of *A*. *disparis*, we discuss potential factors contributing to the evolution of fatal conflict in this species.

## Introduction

In most animal species, aggressive behaviour is important for the acquisition of resources such as food, territory and mates. However, individuals of many species tend to avoid conflict escalation because escalated conflict is energetically costly and potentially damaging^1^. Some species (e.g., mites, spiders, entomopathogenic nematodes, Hymenoptera insects), however, exhibit escalated conflict behaviour^2–5^. It has been postulated that as critical resources become limited, the benefits of winning far outweigh the potential costs of conflict and may lead to extreme forms of fighting that can be fatal^6,7^.

Because access to females is important to males, being directly related to male fitness^8^, male competition for mating opportunities is observed in almost all species^9,10^. In Hymenoptera insects, male competition for mating opportunities is common, especially in quasi-gregarious and gregarious species that mate near or at the emergence site^11^. However, there are few reports of species that engage in lethal male-male combat for mating opportunities. Such species include *Cardiocondyla* ants, fig wasps, and most *Melittobia* species^12–15^. In these species, the occurrence and intensity of combat is influenced by the value of the resource^6,16^, body size^17–19^, competitor density^8,13,20–22^, and relatedness^12,23^.

*Anastatus disparis* (Hymenoptera: Eupelmidae) is a quasi-gregarious egg parasitoid of Lepidoptera in which the hosts are spatially clustered [e.g., egg masses of *Lymantria dispar* (Lepidoptera: Erebidae) and clutches of *Antheraea pernyi* (Guerin-Meneville) eggs], but there is only one parasitoid per host^24,25^. Under natural conditions and in the laboratory, multiple females have been observed to lay eggs on the same patch^24,26^. *A*. *disparis* is protandrous, with males emerging before females. When reared at 28 °C with a photoperiod of 14 L:10 D, the development time was found to be 19–22 days for males and 20–28 days for females^27^. Similar to *Anastatus* sp, most males stay at or near the host after eclosion. Adults can mate immediately after females emergence, near the emergence site, then females search other host for parasitizing^24,28,29^. Among wasps reared from *A*. *pernyi* eggs, the sex ratio was female-biased, with 10–20% of individuals being male^27,28^. And 41–64% of the males eclosed on the first day when reared on *A*. *pernyi* eggs^27^. Furthermore, the number of males reared on field-collected egg mass of *L*. *dispar* ranges from 4 to 28 males^30^. In *A*. *disparis*, we observed prevalent male fighting behaviour. Therefore, a series of experiments were designed and conducted to study the factors that influence fight intensity and fighting characteristics. In all experiments, we used injury and death as proxies to quantify fighting intensity^8,13,20–22^.

## Results

### Competitor density and relatedness

**Figure 1:**
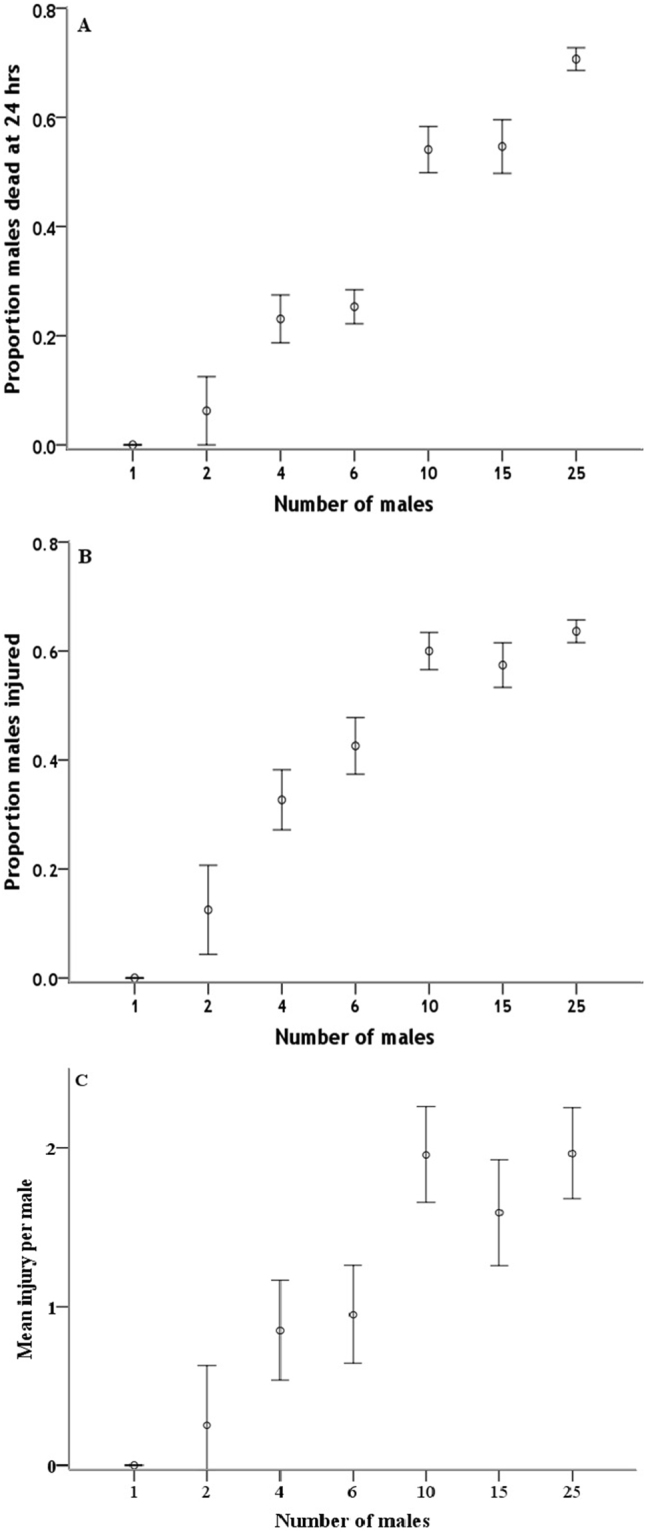
Influence of group size on fight intensity in *A*. *disparis*. Fight intensity was measured as the proportion of males dead (**A**) or injured (**B**) or mean injury per male (**C**) after 24 hours. The error bars indicate standard errors.

Competitor group size had a significant effect on the proportions of dead (Fig. 1A; *F*_6,128_ = 44.73, *p* < 0.001) and injured (Fig. 1B; F_6,128_ = 8.92, *p* < 0.001) males and on the mean injury per male (χ^2^ = 73.05, *df* = 6, *p* < 0.01).

**Figure 2:**
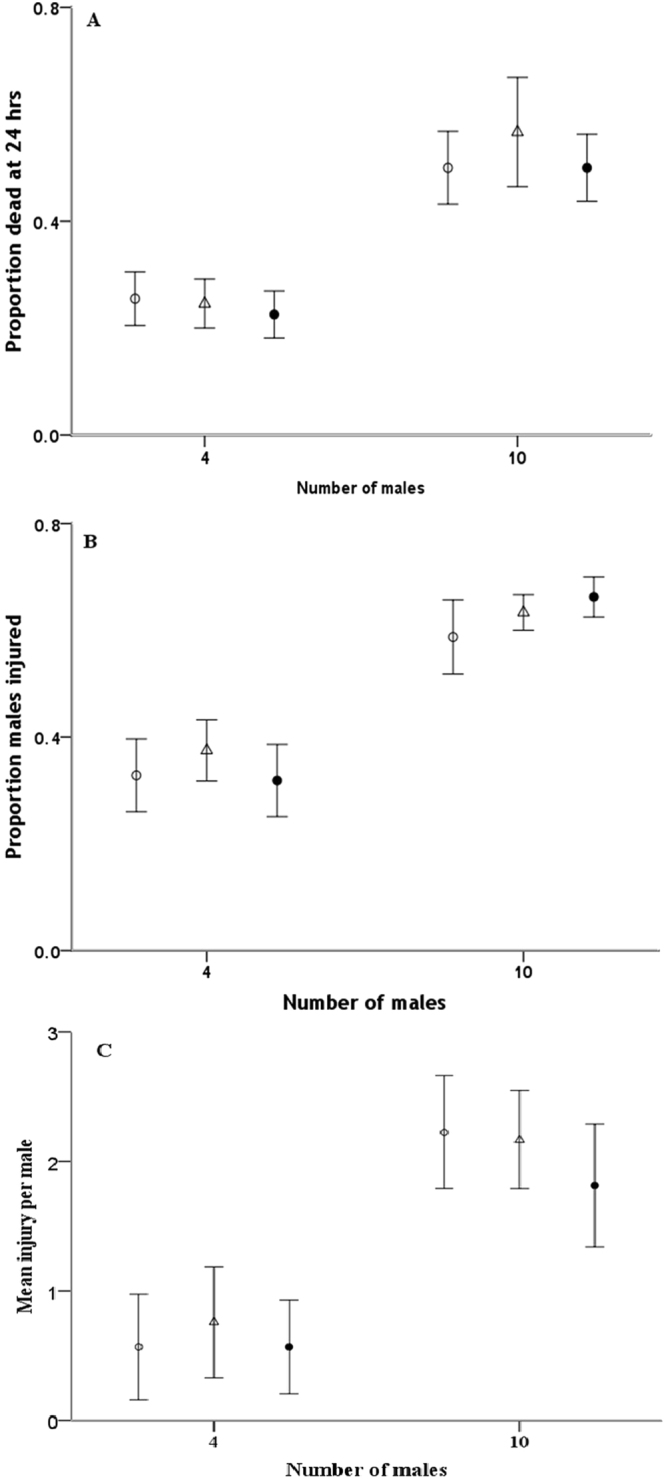
Influence of relatedness (mixed relatedness (open circles), high relatedness (open triangle), and **partial relatedness** (closed circles)) in groups of 4 or 10 male competitors on fight intensity in *A*. *disparis*. Fight intensity was assessed by the proportion of males dead (**A**) or injured (**B**) or mean injury per male (**C**) after 24 hours. The error bars indicate standard errors.

When *A*. *disparis* males were allowed to interact with both nonrelatives and relatives in an arena, we found that the proportions of dead and injured males after 24 hours increased significantly as the group size of competitors increased (dead male proportion, *F*_1,82_ = 20.49, *p* < 0.001; injured male proportion, *F*_1,82_ = 9.50, *p* < 0.05), but there was no significant effect of male relatedness (dead male proportion, *F*_2,82_ = 0.91, *p* > 0.05; injured male proportion, *F*_2,82_ = 1.05, *p* > 0.05) (Fig. 2). Similarly, the mean injury per male increased significantly as the group size of competitors increased (χ^2^ = 21.27, *df* = 1, *p* < 0.01), with no significant effect of male relatedness (χ^2^ = 3.99, *df* = 2, *p* > 0.05).

### Female presence

**Figure 3:**
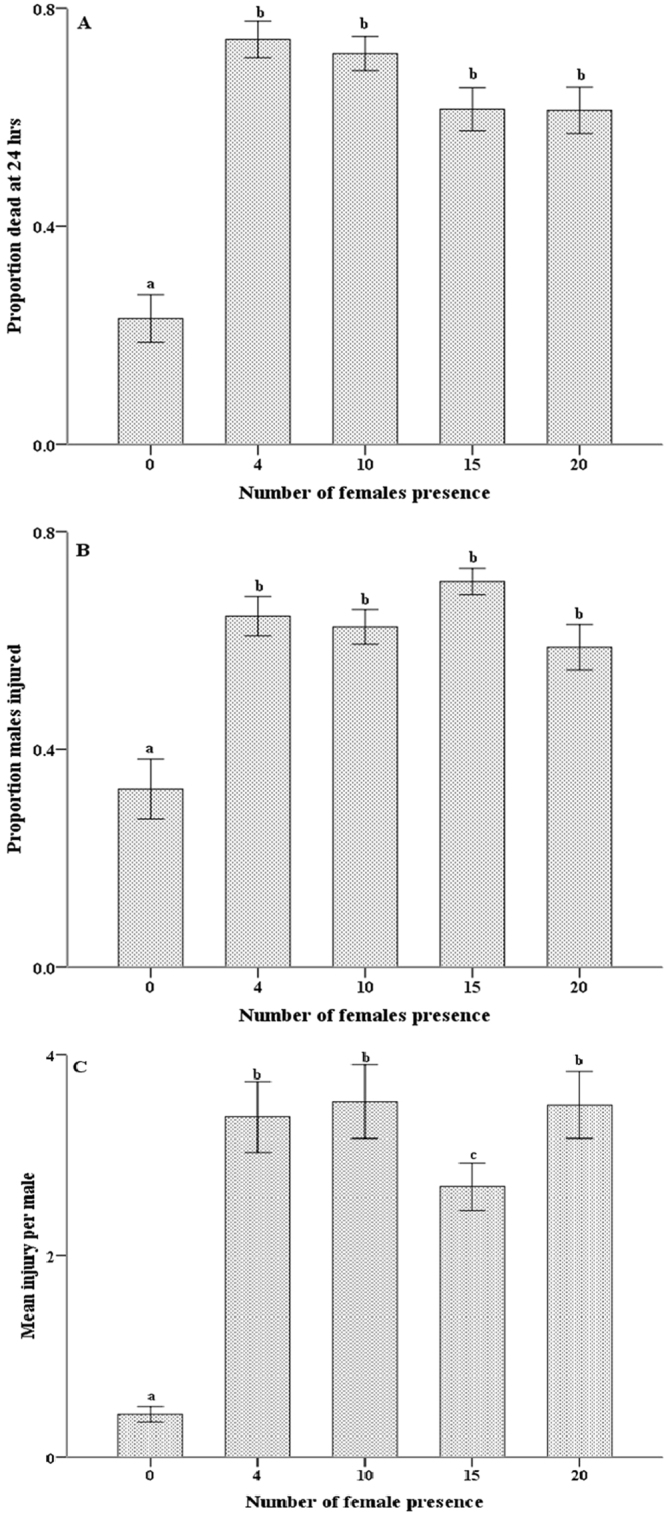
Influence of female presence and number on male fight intensity in *A*. *disparis*. Fight intensity was measured by the proportion of males dead (**A**) or injured (**B**) or mean injury per male (**C**) after 24 hours. Error bars indicate standard errors, and bars with different letters are significantly different at *P* < 0.05.

Female presence significantly affected male aggression, as measured by the proportions of dead (Fig. 3; *F*_1,69_ = 45.73, *p* < 0.05) and injured (Fig. 3; *F*_1,69_ = 15.29, *p* < 0.05) males and the mean injury per male (χ^2^ = 38.50, *df* = 1, *p* < 0.01). In the presence of females, female number did not significantly affect the proportion of dead (Fig. 3; *F*_3,57_ = 1.49, *p* > 0.05) or injured (Fig. 3; *F*_3,57_ = 0.43, *p* > 0.05) males but did significantly affect the mean injury per male (χ^2^ = 6.23, *df* = 3, *p* < 0.05).

### Injured male

**Figure 4:**
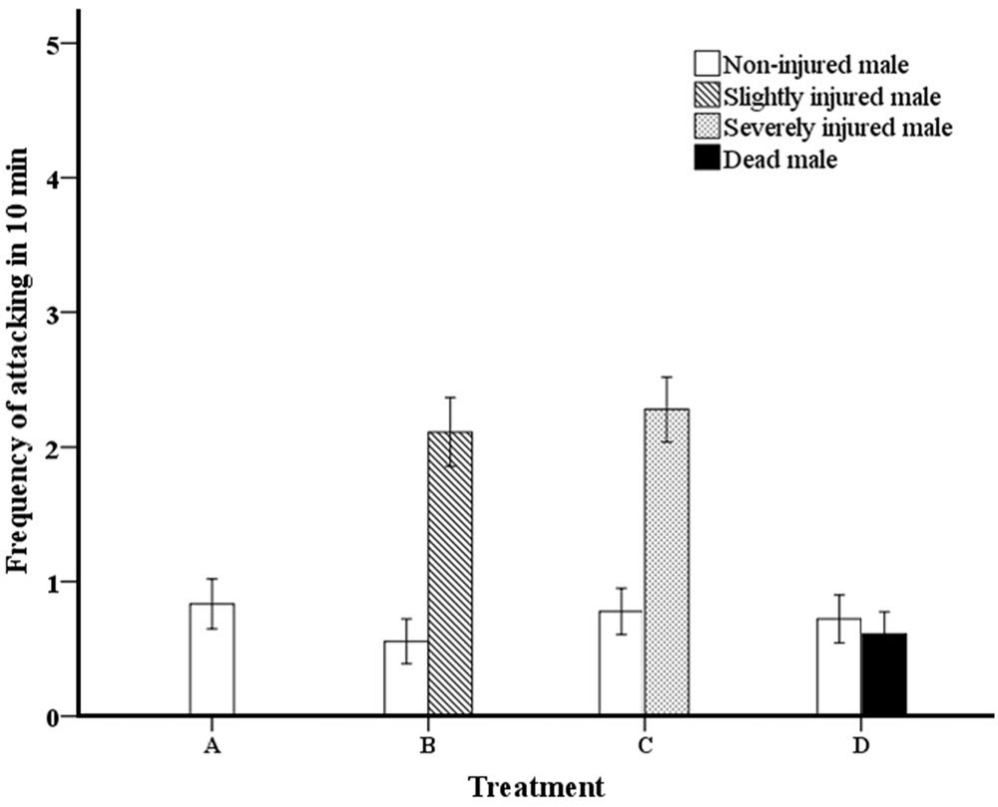
Mean attack frequency in 4 treatments: three uninjured males (**A**), two uninjured males and one slightly injured male (**B**), two uninjured males and one severely injured male (**C**), and two uninjured males and one dead male (**D**). Error bars indicate standard errors.

In general, there were no significant differences in the frequency of attacks between the uninjured males in four treatments (*p* > 0.05). In the treatment with three uninjured males, the frequency of attack was 0.72 ± 0.18 over a 10-min period (Fig. 4. **Treatment A**). In the treatment consisting of two uninjured males and one slightly injured male, the slightly injured male was attacked at a higher frequency than the two uninjured males were attacked by each other (Fig. 4. **Treatment B**; 1-tailed sign test; *p* < 0.05). However, the frequency at which the uninjured males were attacked by the slightly injured male was lower than the attack frequency between each other (1-tailed sign test; *p* < 0.05). In the treatment consisting of two uninjured and one severely injured male, the frequency of attacks between the uninjured males was significantly lower than the frequency at which the severely injured male was attacked (Fig. 4. **Treatment C**; 1-tailed sign test; *p* < 0.05). Severely injured males did not attack other individuals. The uninjured males sometimes also attacked dead individuals; while the frequency of attacks between uninjured males did not significantly differ from the frequency at which dead males were attacked by uninjured males (Fig. 4. **Treatment D**; 1-tailed sign test; *p* > 0.05).

## Discussion

In this study, male-male combat behaviour in *A*. *disparis* was observed to lead to male injury and death. In general, fight intensity was influenced by competitor density. Prior predictions have suggested either a negative^23^ or domed^13^ relationship between fight intensity and competitor density, depending on whether the benefit of winning or the cost of fighting is more influential; an increased linear relationship has also been proposed^8^. Our results showed that the intensity of fighting increased with competitor density (Fig. 1), which may be the result of an increased rate of male encounters^8,13,31^. It also might suggest that each male increased its fighting level in response to increased competitor density.

If males can discriminate kin from non-kin, Hamilton’s theory of kin selection suggests that fight intensity should be lower between closely related competitors than between less related ones and that more altruism should be evident in the former^32,33^. Additional studies have also indicated that limited dispersal would increase local competition among relatives^23,34–36^. However, when *A*. *disparis* males were allowed to interact with both nonrelatives and relatives in an arena, we found no evidence that male competitors adjust their fighting behaviour in response to relatedness (Fig. 3. see also studies in *Melittobia* species^8,37^). This result may be consistent with a comparative study on certain non-pollinating fig wasps, whereby relatedness between males did not influence fighting level^38^. Moreover, as discussed and reviewed in West *et al*.^36,38^, if males recognize relatives, they are expected to adjust their fighting level depending on relatedness. However, if a male cannot recognize relatives, fighting levels are not expected to be influenced by relatedness. Therefore, our results suggest that *A*. *disparis* may be unable to discriminate kin—an ability that is rarely observed in non-social insect species^8,39–43^. Another possibility is that the degree of relatedness may show little variation^44^. Theory predicts that genetic kin discrimination based on genetic polymorphism will be rare because kin discrimination would reduce genetic diversity if common alleles are frequently recognized^45,46^. In the present study, the use of third-generation lines from a potentially inbred or genetically uniform colony might have led to little variation in relatedness among treatments. Molecular methods could be used to quantify relatedness in future studies, and distinct strains, ideally collected from the wild, would be useful.

As mating is directly related to male fitness, access to female mates is important to males^9–11^, and the occurrence and intensity of combat is influenced by the value of the resource, such as mating opportunity^6,16^. Our results showed that the intensity of male fighting was greater under female presence (Fig. 3). This finding suggests that males may escalate conflict to gain the valuable resource of mating opportunities. It is likely that in environments with few mating opportunities, the potential benefits of winning can exceed the costs of fatal combat^6,13,47^. However, as the number of females present in the arena increased (as a proxy for the number of mating opportunities, Fig. 3), fighting intensity (proportion of dead and injured) was still at a high level and did not change much. As observed in some other parasitoid species (e.g., *Nasonia vitripennis* ^48^), individual *A*. *dispar* males can mate with more than 10 females in 24 hours (Liu & Hao’s observation). And mating is directly related to male fitness^9–11^, therefore, each male may be not expected to adjust their fighting level in response to increased females in an effort to acquire more mating opportunities. Another possible reason is that males lack the ability to estimate the number of females in the environment.

Inconsistent with the lethal attack (e.g., decapitation) that occurs in *Melittobia* species^31,49–51^, a single attack cannot lead to death of an individual in *A*. *disparis*. We found that the frequency of attack by others was higher for injured males than for uninjured males; in addition, even dead males were sometimes attacked. These results suggest that injured males may be vulnerable to groups and to frequent and repeated attacks by competitors. One possible reason for these findings is that the capacity of injured males (and dead males) to evade attack is decreased due to reduced mobility. Alternatively, weak individuals may generally receive more aggression within a range of contexts; further work is needed to test this possibility. Death from combat in *A*. *disparis* appears to have been the result of the cumulative effects of injuries sustained from frequent and repeated attacks.

Because of damage to the organism, most species avoid conflict escalation^1^, but this condition was not observed in *A*. *disparis*. First, the mating system perhaps contributes to the evolution of extreme conflict. Although there is only one *A*. *disparis* parasitoid per host, the hosts tend to be spatially clumped^24,25^, which leads to mating^24,28,29^ and male competition for mates nearby the emergence site. When males compete for mates, the females are limited in space, time, or both, and extreme competition and fatal combat behaviours can evolve^8,12,13,23^. Second, the short lifetime of these males (5–8 day) in field^24,26,29^ may lead to low expected-lifetime male mating success^6^. Thus, each successful mating may represent a considerably larger proportion of lifetime reproduction, and in turn, the benefits of winning far outweigh the potential costs of conflict and may lead to extreme forms of fighting that can be fatal^6,7^. Moreover, the vulnerability of injured males may serve as a factor contributing to the evolution of fatal combat behaviours.

## Methods

### Parasitoids and hosts

An *A*. *disparis* colony was established from a population reared on fur-covered *L*. *dispar* egg masses collected from Longhua County, Hebei Province (41°31′N, 117°74′E), China, in March 2012. The colony was maintained on *A*. *pernyi* eggs. The pupae of *A*. *pernyi* are cultivated commercially for their silk; in this study, they were purchased from a farmer in Qinglong Manchu Autonomous County, Qinhuangdao City, Hebei Province, China. Before acquiring the host eggs, the pupae of *A*. *pernyi* were maintained at 26–28 °C prior to adult emergence. Upon emergence of the females, they were sacrificed and dissected, and the eggs were removed from their abdomens. The eggs were maintained at 0 °C prior to use for no longer than 60 days^52^.

To reduce male relatedness in each experiment, third-generation males were obtained by separating the grandmothers during oviposition. Because sex determination in *A*. *disparis* is haplodiploid, virgin females lay unfertilized eggs that develop into males^27,28^. To obtain males, we housed virgin females with 20–30 *A*. *pernyi* eggs for oviposition and then incubated the hosts at 26–28 °C. After 24 hours, we isolated the hosts individually in polyethylene tubes (height: 7.5 cm; diameter: 1 cm) whose openings were covered by a cotton ball to prevent any aggressive male-male behaviour before the experiment began. After each virgin female’s androgenesis, they were mated and again supplied with 20–30 hosts and allowed to oviposit for 24 hours. Then, the hosts were individually isolated in polyethylene tubes to prevent mating behaviour. After approximately 18 days^27^, females and males were checked daily and grouped by eclosion date. For the subsequent experimental replicates, we selected insects that had emerged within the same 24-hour period.

### Experiment 1: Competitor density

To investigate whether the intensity of fighting is influenced by the number of competitors^8,13,23^, we established 7 biologically relevant levels of male density: 1, 2, 4, 6, 10, 15 and 25 males (see above for details of *A*. *disparis* biological characteristics) with 15, 8, 17, 26, 27, 22, and 20 repetitions, respectively. Groups of age- and relatedness-matched males of each density were placed into cylindrical arenas (height: 5 cm, diameter: 10 cm) at 28 °C for 24 hours. For each density, males that emerged within the 24-hour period and from a single female were selected for this experiment. We then isolated all the males (dead, injured and healthy) individually in polyethylene tubes (height: 7.5 cm; diameter: 1 cm). The number of dead and injured males was recorded using a microscope; we also scored each male injury visible with the microscope according to a scale from 0–7 (e.g., the loss of an antennae scored 0.5 points) adapted from Murray^13,20–22^. We then calculated the mean injury per wasp and the proportions of male dead and injured for each arena.

### Experiment 2: Relatedness

In each arena containing 4 or 10 males, we varied the relatedness of the male competitors among 3 treatments: “high relatedness”, in which both males were from a single female (14 and 16 repetitions for the 4- and 10-male density levels, respectively); “mixed relatedness”, in which the males were from unrelated females (13 and 15 repetitions for the 4- and 10-male density levels, respectively); and “partial relatedness”, in which an equal number of males came from 1 of 2 females, meaning that each male was relatively less related to the males from the unrelated female and more related to the males from the same female (15 and 15 repetitions for the 4- and 10-male density levels, respectively). We also calculated the mean injury per wasp and the proportions of male dead and injured for each arena.

### Experiment 3: Female presence

*A*. *disparis* is protandrous; at 28 °C, 41–64% of the males emerge on the first day followed by male and female emergence over 2–3 days^27^. We investigated the effect of female presence on male fighting intensity by varying the number of females present (0, 4, 10, 15 and 20 females, with 12, 14, 14, 19 and 12 repetitions, respectively) with groups of 4 age-matched males. We selected virgin females that had emerged within the 24-hour period for this experiment. The mean injuries per wasp and the proportions of dead and injured males in each arena were calculated.

### Experiment 4: Characteristics of lethal combat

In lethal combat, death may result from a single lethal attack, such as decapitation in *Melittobia* ^31,49–51^. Although *A*. *disparis* males were frequently observed to die following combat, especially under high competitor density (results of **Experiment 1**), deaths were not observed to occur due to single lethal attacks. In combat, most competitors collided with each other and severed their opponents’ limbs using their mouthparts. Based on our casual observations, injured males appeared to be attacked more often and more readily than did uninjured males. Therefore, we hypothesized that injured males are vulnerable in groups and are frequently and repeatedly attacked by competitors, which eventually results in death. To test this hypothesis, we compared the frequency of attack by uninjured males between injured and uninjured males. We considered the degree of injury, i.e., severe or slight, as well as death. Non-moving males were considered to be severely injured. This level of injury generally involves the loss of two or more legs or more serious injuries. Males that had lost part or all of a single leg or antenna were considered slightly injured. Three males (comprising two uninjured males and one slightly injured, severely injured or dead male or comprising three uninjured males; 18, 13, 18, and 17 repetitions, respectively) were placed into cylindrical arenas (height: 5 cm, diameter: 10 cm) at 28 °C. Then, we recorded the number of times each individual was attacked (e.g., male-male contact) over a 10-min period.

### Statistical analysis

As proportional data often have non-normally distributed error variance and unequal sample sizes, the generalized linear model was performed with binomial errors and using a logit link function. Proportional data can also be overdispersed, leading to overestimation of significance. If the heterogeneity factor (HF = residual deviance/residual degrees of freedom) is <4, the data can be scaled and the significance tested using the *F* distribution to correct for overdispersion^53^. In **Experiments 1 and 3**, we used the generalized linear model to test for the effect of male group size and female number on the proportion of males dead or injured after 24 hours. In **Experiment 2**, the generalized linear mixed model was applied to test for effects of male group size and competitor relatedness on mortality and injury. We used the measures of fight intensity as the response variables for each model, including group size and relatedness as fixed effects. Interactions are presented only where significant at the level of *p* < 0.01; this criterion for significance is recommended when testing interactions^53^. In addition, the data for scores (mean injury per male) was analysed by the nonparametric method using the Kruskal-Wallis H test. In **Experiment 4**, male-male attack frequency in the same treatment was analysed using sign tests, and the Kruskal-Wallis H test was also used to analyse the attack frequency between different treatments. All statistical analyses were performed using SPSS software (version 20).
